# Selective Brain Cooling Reduces Water Turnover in Dehydrated Sheep

**DOI:** 10.1371/journal.pone.0115514

**Published:** 2015-02-12

**Authors:** W. Maartin Strauss, Robyn S. Hetem, Duncan Mitchell, Shane K. Maloney, Leith C. R. Meyer, Andrea Fuller

**Affiliations:** 1 Brain Function Research Group, School of Physiology, Faculty of Health Sciences, University of the Witwatersrand, Johannesburg, South Africa; 2 Department of Environmental Sciences, College of Agriculture and Environmental Sciences, University of South Africa, Johannesburg, South Africa; 3 School of Anatomy, Physiology, and Human Biology, University of Western Australia, Crawley 6009, Australia; INIA, SPAIN

## Abstract

In artiodactyls, arterial blood destined for the brain can be cooled through counter-current heat exchange within the cavernous sinus via a process called selective brain cooling. We test the hypothesis that selective brain cooling, which results in lowered hypothalamic temperature, contributes to water conservation in sheep. Nine Dorper sheep, instrumented to provide measurements of carotid blood and brain temperature, were dosed with deuterium oxide (D_2_O), exposed to heat for 8 days (40◦C for 6-h per day) and deprived of water for the last five days (days 3 to 8). Plasma osmolality increased and the body water fraction decreased over the five days of water deprivation, with the sheep losing 16.7% of their body mass. Following water deprivation, both the mean 24h carotid blood temperature and the mean 24h brain temperature increased, but carotid blood temperature increased more than did brain temperature resulting in increased selective brain cooling. There was considerable inter-individual variation in the degree to which individual sheep used selective brain cooling. In general, sheep spent more time using selective brain cooling, and it was of greater magnitude, when dehydrated compared to when they were euhydrated. We found a significant positive correlation between selective brain cooling magnitude and osmolality (an index of hydration state). Both the magnitude of selective brain cooling and the proportion of time that sheep spent selective brain cooling were negatively correlated with water turnover. Sheep that used selective brain cooling more frequently, and with greater magnitude, lost less water than did conspecifics using selective brain cooling less efficiently. Our results show that a 50kg sheep can save 2.6L of water per day (~60% of daily water intake) when it employs selective brain cooling for 50% of the day during heat exposure. We conclude that selective brain cooling has a water conservation function in artiodactyls.

## Introduction

Several mammal species, particularly artiodactyls (such as sheep, goats and antelope), use the carotid rete to lower hypothalamic temperature below arterial blood temperature, a process termed selective brain cooling [1]. Typically, in resting artiodactyls, when carotid blood temperature exceeds a threshold of 38.5–39.0°C [2], selective brain cooling is implemented, and brain temperature increases at a slower rate than does carotid temperature. However, selective brain cooling is not mandatory at high body temperatures and can be switched off by high sympathetic nervous system activity [3, 4]. Sympathetic nervous system activity influences blood flow to the cavernous sinus, which envelopes the carotid rete with cool blood, through the constriction of nasal mucosal blood vessels and the closure of arteriovenous anastomoses [5] located in the nasal cavity [6]. Variation in sympathetic tone between individuals of the same species appears to account for high inter-individual variability in the use of selective brain cooling [7, 8], which can be quantified by assessing the magnitude of the difference between brain and carotid blood temperature, the proportion of time spent with brain temperature lower than carotid blood temperature, and the threshold temperature for selective brain cooling [9].

Selective brain cooling initially was thought to protect a thermally vulnerable brain during hyperthermia [10, 11]. But such a protective role for selective brain cooling has been found wanting [12]. It is now thought that selective brain cooling plays a role in conserving body water by cooling the temperature sensors in the hypothalamic region of the brain, thereby reducing heat loss drive [1, 9, 12–14]. Indeed, when dehydrated, Bedouin goats [15] and Dorper sheep [8] showed enhanced selective brain cooling, both in terms of magnitude and the frequency of use. However, the higher carotid blood and brain temperatures recorded in the Bedouin goats during dehydration meant that the increased selective brain cooling also could be explained by the higher core temperature compared to the period of euhydration [8]. Conversely, the carotid blood temperature in the sheep did not increase during dehydration, yet selective brain cooling was still enhanced, showing that the increase in selective brain cooling was a function of dehydration, not simply elevated core body temperature [8]. Studies in free-ranging antelope also support the idea that selective brain cooling is enhanced in arid environments independently of heat exposure. Arabian oryx had a higher magnitude, higher frequency of use, and lower threshold of selective brain cooling in dry environments than when they had free access to water, despite ambient temperatures being similar [9].

These studies provide circumstantial evidence in support of the notion that selective brain cooling serves a water conservation function. To date, only a single laboratory study has investigated the relationship between selective brain cooling and water use. Goats, in which extra-corporeal heat exchangers were used to independently manipulate carotid blood and brain temperatures, had lower respiratory evaporative heat loss when the brain was cooled artificially and the body core heated, than when the brain was not cooled [2]. Here we investigate, for the first time, whether water savings accrue in artiodactyls spontaneously using selective brain cooling. Dorper sheep were fitted with temperature probes that provided continuous, real-time, temperature measurements from the brain and carotid artery. We measured water turnover using the washout rate of deuterium oxide. We hypothesized that if selective brain cooling does play a role in water conservation it would lead to reduced water turnover in those sheep showing greater selective brain cooling.

## Methods

### Animals

Nine adult Dorper ewes (Dorset x Persian *Ovis aries*, initial body mass 60 ± 15 kg, mean ± SD) were used. The mean fleece length of the ewes at study termination, as measured at the shoulder and on the hip, was 18.0 ± 3.8 mm and 15.4 ± 2.2 mm, respectively. The sheep were housed indoors in a single pen at an ambient temperature of ~22°C on a 12h/12h light/dark cycle (lights on from 06:00–18:00) for two weeks before implantation surgery. Lucerne, *Eragrostis* teff and water were available *ad libitum*, while commercial sheep pellets (Epol, Johannesburg, South Africa) were provided to the sheep once per day. The water content of the food was estimated to be no more than 5%. Fresh bedding (dry straw) was provided daily after the holding pen was cleaned.

### Ethics statement

The Animal Ethics Screening Committee of the University of the Witwatersrand approved all of the procedures (protocol no. 2008/55/04).

### Surgery

In the holding pen the sheep were given diazepam (0.3mg kg^-1^ Valium, Roche, Nutley, NJ, USA) intra-muscularly (IM). About 15min later butorphanol (0.1mg kg^-1^, Torbugesic, Fort Dodge, Kempton Park, South Africa) and ketamine (2mg kg^-1^, Anaket, Bayer Animal Health, Isando, South Africa) were administered intravenously (IV) and the sheep were transported to a nearby sterile theatre. At the theatre, the sheep were maintained in sternal recumbency with the help of sandbags. Following intubation, general anesthesia was maintained with 2–3% isoflurane (Isofor, Safe Line Pharmaceuticals, Johannesburg, South Africa) in oxygen. The surgical incision sites were shaved and sterilized with chlorhexidine gluconate (Hibitane, Zeneca, Johannesburg, South Africa). Each sheep received a long-acting antibiotic (0.1ml kg^-1^, IM, penicillin, Peni LA Phenix, Virbac Animal Health, Centurion, South Africa), an analgesic (0.01mg kg^-1^, sub-cutaneously (SC), buprenorphine, Temgesic, Schering Plough, Isando, South Africa), a non-steroidal anti-inflammatory (2.2mg kg^-1^, IM, flunixin meglumine, Finadyne, Centaur Labs, Johannesburg, South Africa), a broad-spectrum parasiticide (2.5ml SC, doramectin, Dectomax, Pfizer Laboratories, Sandton, South Africa), and a multivitamin (Mulitivit injectable solution, Univet Ltd, County Cavan, Ireland) injection. To anesthetize the periosteum and reduce bleeding a 1.5ml cocktail of lignocaine (0.1g, Bayer Animal Health, Johannesburg, South Africa) and adrenaline (Kyron Labs, Johannesburg, South Africa), mixed at a ratio of 10:1, was injected subcutaneously under the scalp where the brain probe would be inserted. Under sterile surgical procedures, we implanted two thermometric radio-telemeters into each animal (see below). We measured respiratory rate (visual observation), heart rate, peripheral arterial oxygen saturation (Nonin Handheld Pulse Oximeter, Plymouth, MN, USA) and rectal temperature (Cardell 9400, Midmark Corporation, Ohio, USA) throughout the ~2 hour surgical procedure. Implanted devices were covered with inert wax (Sasol, Johannesburg, South Africa) and dry-sterilized using formaldehyde vapor for at least 24 hours before implantation.

### Temperature measurement

Ruggedized glass-coated bead thermistors (bead diameter 0.3mm; ABOE3-BR11KA103K-L10, Thermometrics Corporation, Northridge, CA, USA) in sealed guide tubes specific for each implantation site were used to measure temperatures in the brain and carotid artery. For brain temperature measurements, a cellulose acetate butyrate guide tube (length 44mm, OD 3.2mm, ID 1.6mm; World Precision Instruments, Sarasota, FL, USA) sealed with a stainless-steel cap at the tip was positioned near the hypothalamic region of the brain. The guide tube was inserted via a 2.0mm hole drilled through the cranium, ~3.0mm to the left of the mid-line of the skull, at previously determined anatomical markers [16]. The guide tube was connected to a polyvinylchloride head plate (20 x 10 x 5mm). The head plate was attached to the skull with two bone screws and covered with skin. A polytetrafluoroethylene (PTFE) coated coaxial cable (150mm long, OD 3mm, Belden, Richmond, VA, USA), connected to the thermistor in the head plate, was extended subcutaneously over the skull to a radio-telemeter (Africa Wildlife Tracking, Pretoria, South Africa) specifically developed for our purposes. The radio-telemeter, with dimensions ~70 x 50 x 30mm and mass of 100g when covered with wax, was placed subcutaneously, caudal to the base of the left ear.

To measure carotid arterial blood temperature, a bead thermistor, inserted into a blind-ended, thin-walled PTFE tube (OD 1.35mm, ID 0.97mm; Straight Aortic Flush 4F Catheter, Cordis, The Netherlands) was inserted 80mm into the left common carotid artery, towards the heart. It was secured in position, midway along the length of the neck, with a purse-string suture in the artery wall. Outside the artery, the PTFE tube was sealed onto a PTFE-coated coaxial cable (150mm long, OD 3mm, Belden, Richmond, VA, USA), connecting the thermistor to a second radio-telemeter, identical to that used for brain temperature measurement. Because all the transmitting units were implanted there was no external instrumentation on the animals.

The temperature sensors had a measurement range of 34°C to 50°C and a resolution of 0.03°C. They were calibrated against a high-accuracy thermometer (Quat 100, Heraeus, Hanau, Germany) in an insulated water bath to an accuracy of better than 0.05°C. A single receiver/transmitting unit, which logged temperature data from all animals at 5-minute intervals, was placed ~2m above ground level, and less than 8m away from any animal at any given time during the study. Logged temperature data were also transmitted to an internet server, via GPRS, at hourly intervals, with all temperature records accessible in near-real time.

### Experimental procedure

**Fig 1:**
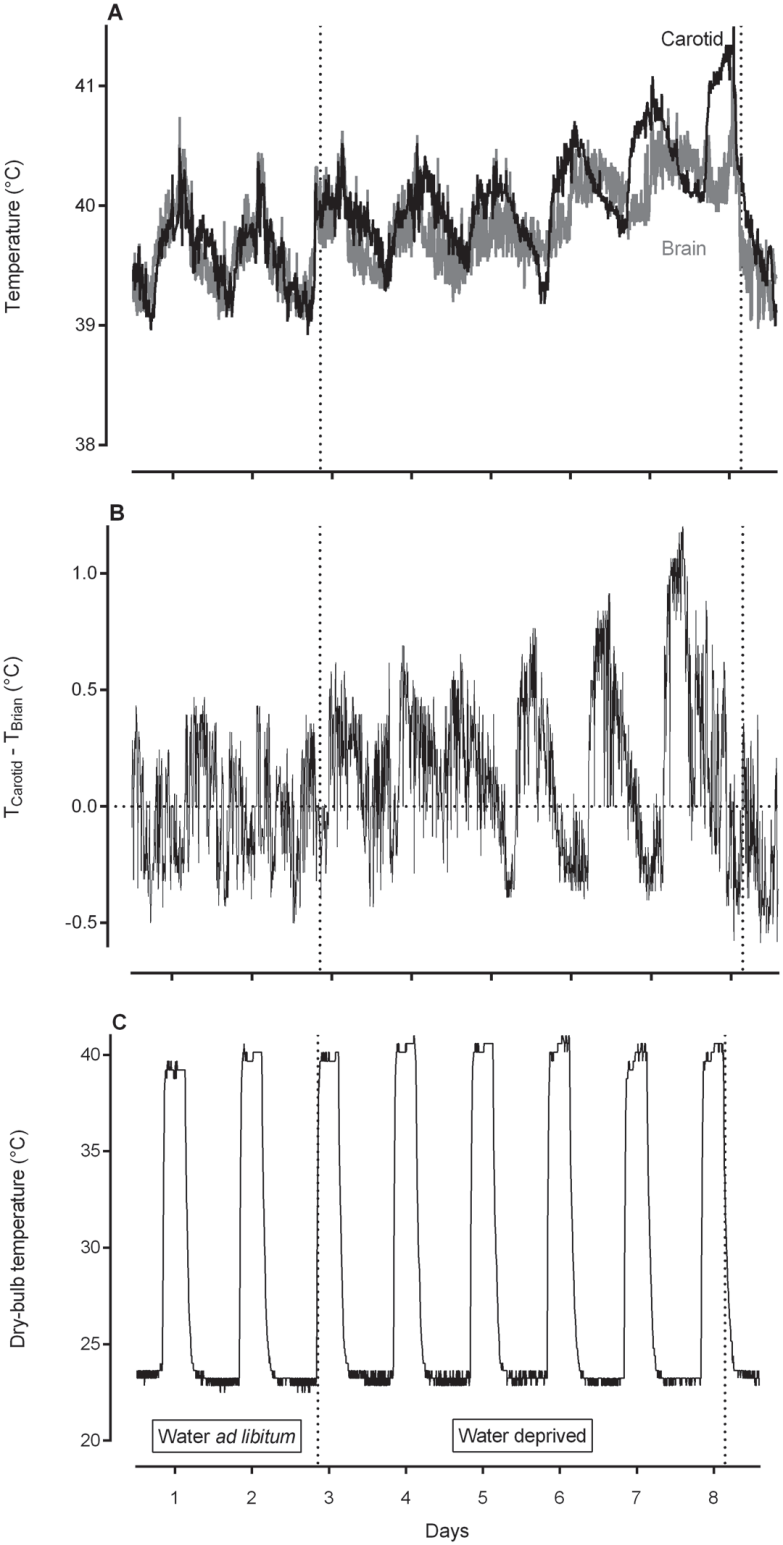
Carotid blood (black) and brain (grey) temperature (A) and the difference between carotid blood and brain temperatures (B; positive values indicating selective brain cooling) at 5-minute intervals for one representative sheep, and daily air dry-bulb temperature profile as recorded in the climatic chamber (C) during an 8-day experimental period. Sheep had *ad libitum* access to drinking water on days 1 and 2. Water was removed on the morning of day 3 and was returned on the afternoon of day 8 (as indicated by the dotted lines). Food was available *ad libitum* throughout the experimental period.

The experimental procedure was repeated twice, once for four sheep and again for five sheep. The sheep were moved into a temperature-controlled climatic chamber (about 7.0m^2^ floor area) ten days after implantation surgery, and allowed to acclimatize to their new environment for two days (12h/12h light/dark cycle, lights on at 06:00, temperature ~22–24°C, relative humidity ~60%). Following acclimatization to the climatic chamber, the sheep were allowed a further two days to acclimatize to the temperature and relative humidity regime that would prevail during the study: heat exposure (40°C; relative humidity ~40%) for a total of six hours per day (09:00–15:00), ~22–24°C, ~60% relative humidity for the remainder of the time (Fig. 1C). Food and water were available freely during the four-day acclimatization period. On the first two days of the study (day 1 & 2), the animals had free access to food and drinking water (as in their holding pen). Drinking water, but not food, was removed on the morning of day 3 (~09:00) and was returned on the afternoon of day 8 (~16:00) to each animal after it was individually weighed. Animals were weighed every afternoon at about 15:00.

We measured water turnover (L day^-1^), the sum of water influx (food and metabolic water) and water loss (change in total body water), by dilution of the stable hydrogen isotope deuterium oxide, over the five days of water deprivation and heat exposure. At the time of water removal on day 3, a 4ml blood sample was collected from the right jugular vein of each sheep to determine background concentrations of deuterium oxide. Each sheep then was injected with 0.05ml kg^-1^ deuterium oxide (D_2_O, 99.8 at%; Merck & Co. Ltd, Rahway, NJ, USA) IM, based on body mass measured on the previous afternoon. The blood sample collection and deuterium injection procedures were completed for all sheep within ~15 minutes of water removal. Following equilibration of the deuterium oxide in the body water pool (~ 8 hours later), another (enriched) 4ml blood sample was collected from the right jugular vein. Using the same procedures, we collected a blood sample every afternoon immediately following heat exposure from each sheep on days 4–7 of the experimental period. Shortly before heat exposure on the morning of day 8 (~08:30, 5^th^ day of water deprivation), a background blood sample was taken, and sheep again received 0.05ml kg^-1^ deuterium oxide IM based on the body mass measured on the previous afternoon. This second injection of deuterium oxide allowed for a measure of total body water volume at the end of the water deprivation period to allow for the calculation of change in total body water over the period of water deprivation. A final blood sample was collected ~8 hours later (i.e. ~15:00 on afternoon of day 8), following equilibration of the deuterium oxide in the body water pool and before drinking water was returned to the sheep.

The blood samples were collected in 4ml heparinized vials (Vacutainer, BD-Plymouth, UK), and placed in ice until centrifuged (Wifug Ltd, Bradford, England) at 6000g for 10 min. Plasma was separated and stored in 2ml vials (cryogenic vials 430489, Corning Inc., NY, USA) and frozen at -4°C for later analysis. A calibrated osmometer (Vapro Model 5600, Wescor Inc., Utah, USA) was used to determine the osmolality of every plasma sample. Pure water was extracted from the plasma samples through cryogenic vacuum distillation [17]. The deuterium concentration of the pure water samples was measured on a liquid water isotope analyzer (LWIA-24d, Los Gatos Research, Mountain View, CA, USA), normalized against an international reference, Vienna Standard Mean Ocean Water (V-SMOW). Individual water samples were analyzed five times and the mean concentration calculated. A comprehensive description of the underlying theory, the available methods and calculations are provided elsewhere [18]. Briefly, we used a two sample method to determine body water turnover, where the elimination rate of the deuterium oxide was calculated through the slope of a regression of the log-transformed concentration plotted against time [18]. The natural exponential function of the y-intercept of the regression line was used to estimate the isotope distribution space. We estimated total body water by dividing the known dose of injected deuterium oxide (ml) by the difference between the natural exponential function of the y-intercept (isotope distribution space) and the background enrichment (ppm) of deuterium oxide. The influx of water (through metabolism and preformed water in the diet) was determined from the deuterium oxide dilution rate over the 5-day period, when sheep did not have access to water, multiplied by the total body water.

### Data analyses

The original 5-minute body temperature records from each sheep were used to find the absolute minimum and the absolute maximum carotid blood and brain temperatures each day, and to calculate the mean, standard deviation and amplitude (difference between absolute maximum and absolute minimum temperatures) for each individual. Selective brain cooling was calculated as the difference between carotid blood temperature and brain temperature, with positive values representing selective brain cooling. We categorized the 48h period before water removal and the 48h period before returning water to the sheep as periods of “euhydration” and “dehydration”, respectively. We used paired Students t-tests to compare temperature data during the periods of euhydration and dehydration. Repeated measures one-way ANOVAs were used to investigate the differences in the daily magnitude of selective brain cooling (calculated as the maximum or mean positive difference between carotid and brain temperature), the proportion of time spent using selective brain cooling (calculated as the proportion of measurements in which the difference between carotid and brain temperature was ≥0.05°C), the threshold temperature (calculated as the temperature at which the regression line of carotid blood versus brain temperature crossed the line of identity) and osmolality throughout the experimental period. We used Pearson correlation analysis to investigate possible relationships between variables. To compare variability in the selective brain cooling response during water deprivation, we correlated the standard deviations of the selective brain cooling magnitude, and the standard deviations of the mean proportion of time spent using selective brain cooling, against days of water deprivation. Also, to investigate the rates at which carotid blood and brain temperatures increased with continued water deprivation we compared the slopes of the linear regression lines of the daily carotid blood and brain temperature profile against days of water deprivation. Data analysis was done using GraphPad PRISM 6. Results are reported as mean ± SD, and P ≤ 0.05 was considered significant.

## Results

Fig. 1A and B show the response of one sheep to the entire experimental protocol. During the first two days, when water was freely available, carotid blood and brain temperatures followed similar profiles and brain temperature frequently exceeded carotid blood temperature. These patterns changed following the removal of water (day 3), with brain temperature clearly decoupling from carotid blood temperature. Peak carotid blood and peak brain temperature increased with continued water deprivation (Fig. 1A), however, the increase in carotid blood temperature was larger than that of brain temperature, leading to increasing separation of the two temperatures and enhanced selective brain cooling. Indeed, the mean difference between carotid blood and brain temperatures was significantly higher during dehydration than during euhydration (t_8_ = 2.83, P = 0.022). Examination of the difference between carotid blood and brain temperature revealed selective brain cooling being used for more of the day (fewer data points below the zero line), and an increased magnitude (greater positive value) of selective brain cooling, as the period of water deprivation progressed (Fig. 1B). The other eight sheep showed similar responses during the experimental protocol.

### Increased carotid and brain temperature with water deprivation

The increased use of selective brain cooling with continued water deprivation (Fig. 1B) was associated with an increase in carotid blood temperature (Fig. 1A). The mean 24h carotid blood temperature was 0.6°C higher during dehydration than during euhydration (t_8_ = 3.48, P = 0.0082; Table 1). Although the minimum carotid blood temperature did not change from euhydration to dehydration (t_8_ = 1.91, P = 0.092), the maximum carotid blood temperature was significantly higher during dehydration than during euhydration (t_8_ = 2.85, P = 0.021). The mean 24h brain temperature also was significantly higher during dehydration than during euhydration (t_8_ = 3.02, P = 0.017). Unlike carotid blood temperature, the minimum brain temperature increased significantly from euhydration to dehydration (t_8_ = 2.43, P = 0.041). The maximum brain temperature also increased significantly from euhydration to dehydration (t_8_ = 2.35, P = 0.047).

**Table 1:** Mean (±SD) daily carotid blood and brain temperatures recorded every 5 minutes and the mean difference between carotid and brain temperatures in nine Dorper ewes during 48h of euhydration and 48h of dehydration.

	Carotid artery temperature		Brain temperature	
	Euhydration	Dehydration	Euhydration	Dehydration
**Mean**	39.36 ± 0.19°C	39.92 ± 0.47°C*	39.36 ± 0.18°C	39.61 ± 0.36°C*
**Minimum**	38.60 ± 0.21°C	38.95 ± 0.63°C	38.76 ± 020°C	38.99 ± 0.43°C*
**Maximum**	40.41 ± 0.39°C	40.96 ± 0.38°C*	40.37 ± 0.31°C	40.56 ± 0.29°C*
**Amplitude**	1.77 ± 0.40°C	1.92 ± 0.55°C	1.60 ± 0.27°C	1.69 ± 0.19°C

* indicates significant differences (P<0.05) between temperatures measured during euhydration and dehydration

To investigate the rate at which carotid and brain temperatures increased with continued water deprivation, we compared the slope of regression lines of the mean and maximum daily temperatures against days of water deprivation. The slope of the linear regression lines of mean daily carotid temperature (*y* = 39.5 + 0.086*x*, r^2^ = 0.97) and mean daily brain temperature (*y* = 39.4 + 0.037*x*, r^2^ = 0.81) against days of water deprivation were significantly different (F_(1,8)_ = 17.37, P = 0.003), with brain temperature having the lower slope. However, the slopes of the regression lines of maximum daily carotid temperature (*y* = 0.093*x* + 40.26, r^2^ = 0.44) and maximum daily brain temperature (*y* = 0.053*x* + 40.19, r^2^ = 0.40) against days of water deprivation did not differ (F_(1,8)_ = 0.43, P = 0.53).

### Increased selective brain cooling with water deprivation

**Fig 2:**
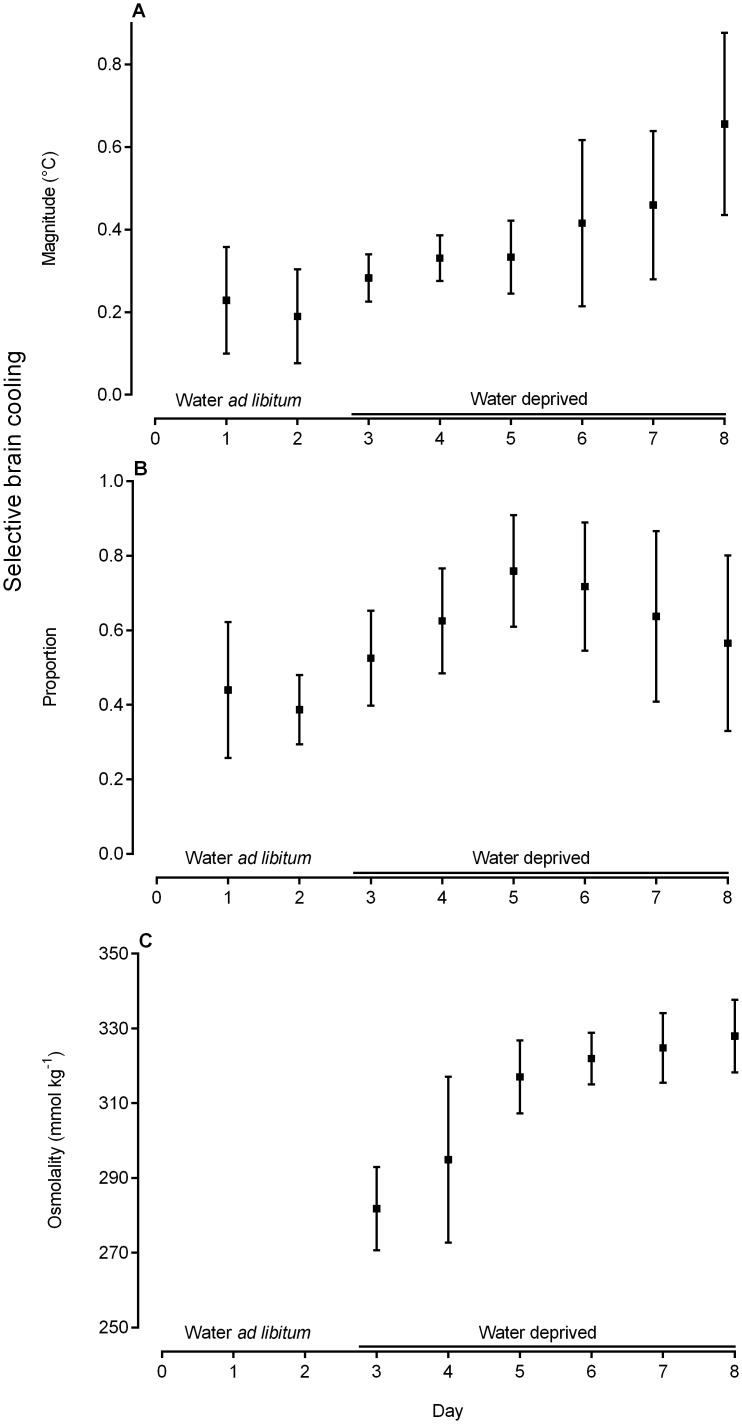
Mean (± SD) of the magnitude of selective brain cooling (A), the proportion of time that selective brain cooling was used (B) and the osmolality (C) as recorded daily for nine sheep over the 8-day experimental period. The five-day period in which water was removed is highlighted by the black line.

The daily magnitude (mean positive difference between carotid blood and brain temperature) of selective brain cooling increased significantly with days of water deprivation (F_(5,8)_ = 10.26, P = 0.0017) from 0.28 ± 0.06°C on day 3 (the day of water removal) to peak at 0.66 ± 0.22°C on day 8 (fifth day of water deprivation; Fig. 2A). The selective brain cooling magnitude was positively correlated with carotid blood temperatures (r^2^ = 0.61, P = 0.013). The mean proportion of time that the sheep spent using selective brain cooling ranged from 52.5 ± 12.8% on day 3 to 76 ± 15% on day 5 (the second day after water removal; Fig. 2B), but did not change significantly over the period of water deprivation, (F_(5,8)_ = 2.23, P = 0.15). Plasma osmolality increased significantly over the period of water deprivation (F_(5,8)_ = 50.09, P < 0.0001), from 282 ± 11mmol kg^-1^ on day 3 to 328 ± 10mmol kg^-1^ on day 8 (Fig. 2C). There was no significant relationship (r^2^ = 0.23, P = 0.34) between the variability of osmolality, as measured by the standard deviation of mean plasma osmolality, and time since water removal. The variability in the use of selective brain cooling by different sheep (Fig. 2A & B), as measured by the standard deviation of the mean magnitude of selective brain cooling (r^2^ = 0.86, P = 0.008) and the standard deviation of the mean proportion of time spent using selective brain cooling (r^2^ = 0.92, P = 0.003), was correlated positively with the duration of the experimental period (days 3 to 8).

**Fig 3:**
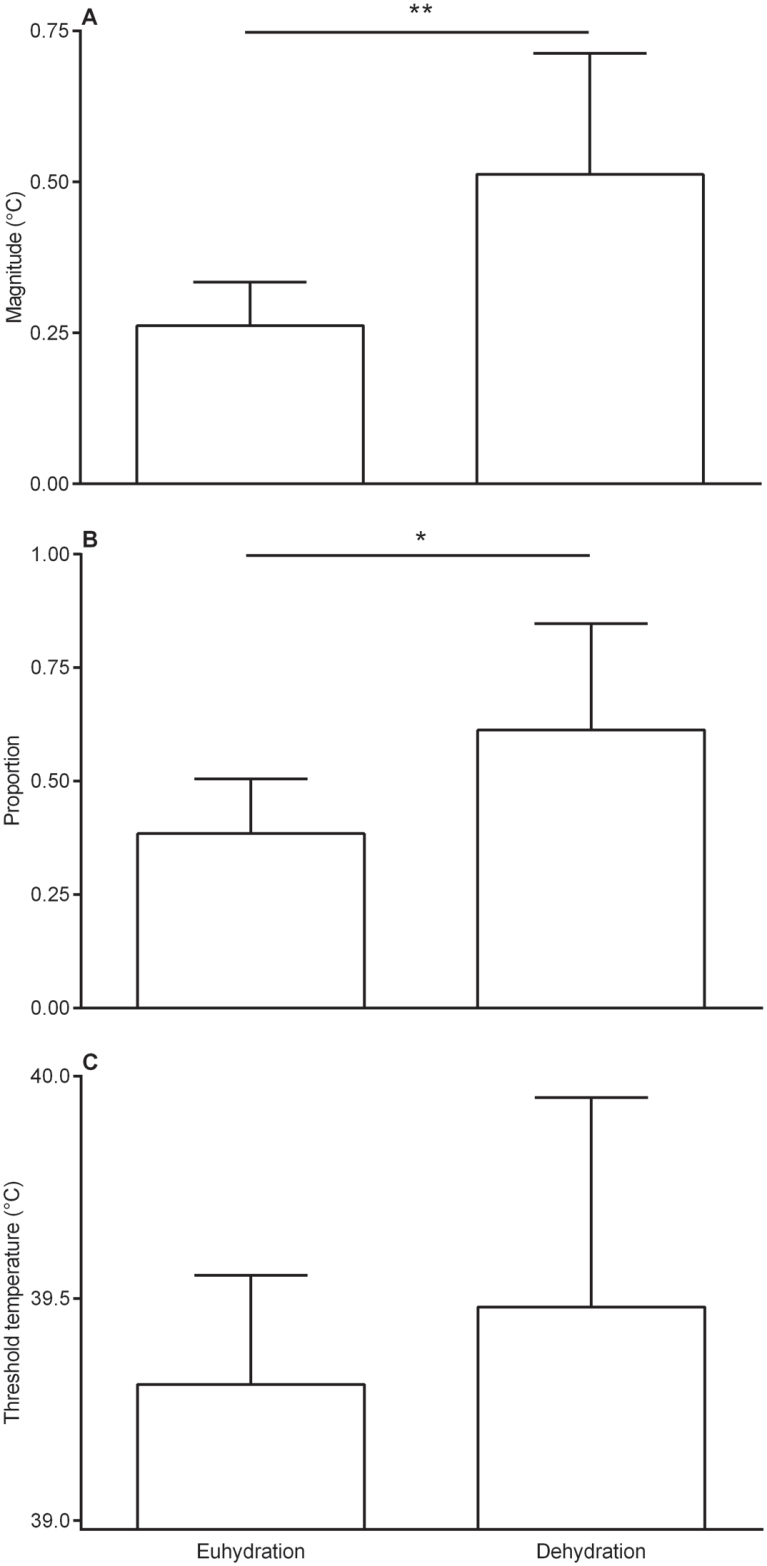
Mean (± SD) of the magnitude of selective brain cooling (A), proportion of time that selective brain cooling was used (B), and the threshold temperature for selective brain cooling (C) as recorded in nine Dorper sheep during euhydration (48 hours before water removal) and dehydration (last 48 hours before returning water). * P<0.05, ** P<0.01 for comparison between euhydration and dehydration.

The mean magnitude of selective brain cooling increased significantly and was twice as high during dehydration (0.51 ± 0.20°C) compared to euhydration (0.26 ± 0.07°C; t_8_ = 3.78, P = 0.0054; Fig. 3A). The maximum magnitude of selective brain cooling was significantly higher during dehydration (1.09 ± 0.37°C) than during euhydration (0.67 ± 0.18°C; t_8_ = 3.32, P = 0.0106). The proportion of time that the sheep, on average, spent using selective brain cooling also increased significantly from their 48h state of euhydration (39 ± 12%) to that of dehydration (61 ± 24%; t_8_ = 2.57, P = 0.033; Fig. 3B). The threshold temperature at which selective brain cooling was initiated did not differ between euhydration (39.31 ± 0.25°C) and dehydration (39.48 ± 0.47°C; t_8_ = 1.18, P = 0.27; Fig. 3C).

### Selective brain cooling and plasma osmolality

**Fig 4:**
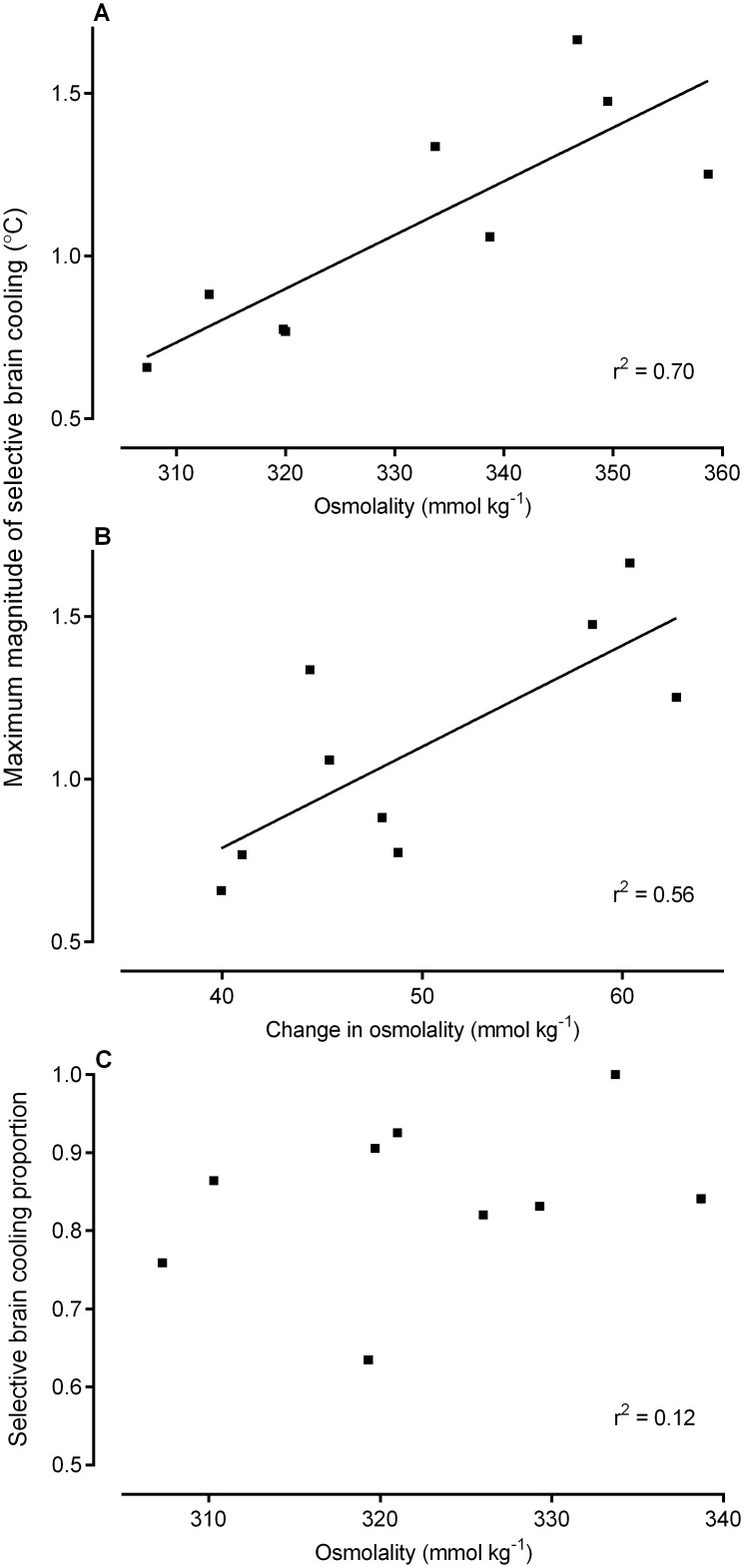
The relationship between the maximum magnitude of selective brain cooling (°C) during the last four days of water deprivation and plasma osmolality as recorded on the day of the maximum (A; *y* = 0.02*x* - 4.38 [± 1.37]), and maximum magnitude of selective brain cooling during the last four days of water deprivation and the change in osmolality (difference between osmolality on day of maximum and osmolality on day 3) (B; *y* = 0.03*x* - 0.46 [± 0.52]), and maximum proportion of time spent using selective brain cooling during the last four days of water deprivation and osmolality on the day of maximum proportion (C) for nine sheep. Each data point represents a single animal.

The body mass of the sheep decreased significantly, by about 16%, from 60 ± 15 kg during euhydration to 50 ± 15 kg (range of change: 7.0–11.6 kg) during dehydration (t_8_ = 17.99, P < 0.0001). Concomitantly, plasma osmolality, on average, showed an increase of 15% (euhydration 287 ± 9 mmol kg^-1^ vs. dehydration 338 ± 15 mmol kg^-1^; t_8_ = 10.96, P < 0.0001). Correlating the maximum magnitude of selective brain cooling recorded per animal during the last four days of water deprivation against osmolality on the day that the maximum magnitude was recorded (i.e. one point per animal) resulted in a significant positive relationship (r^2^ = 0.70, P = 0.005), indicating increased magnitude of selective brain cooling with increased osmolality (Fig. 4A). Correlating the maximum magnitude of selective brain cooling during the last four days of water deprivation against the change in osmolality (the difference between the osmolality on day of maximum selective brain cooling magnitude and the osmolality on day 3) also resulted in a significant positive relationship (r^2^ = 0.56, P = 0.020; Fig. 4B). There was no relationship (r^2^ = 0.12, P = 0.36) between the maximum proportion of time spent using selective brain cooling during a day on the last four days of water deprivation and osmolality on that day (Fig. 4C).

### Selective brain cooling and water loss

**Fig 5:**
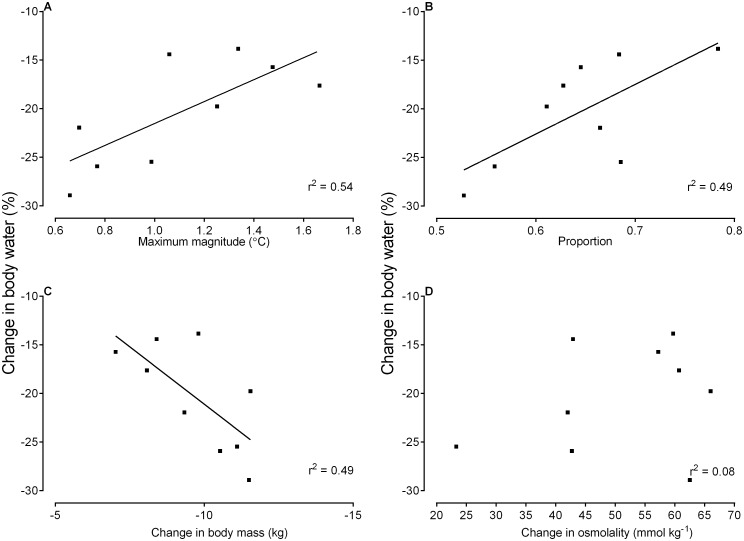
The relationship between change in body water (%) and the maximum magnitude of selective brain cooling (A; y = 50.96x—53.16 [± 12.67]), the proportion of time spent using selective brain cooling (B; y = 11.28x—32.80 [± 4.52]), the change in body mass (C; y = 2.36x + 2.50 [± 9.01]), and the change in plasma osmolality (D; no relationship) as recorded from day 3 to 8 (i.e., five days of water deprivation) in nine sheep. Each data point represents a single animal.

Total body water decreased significantly (range: -1.0–16.9L) from day 3 (41 ± 7L) to day 8 (31 ± 6L; t_8_ = 4.96, P = 0.0011), that is over the five days of water deprivation. The body water fraction (% of body mass), on average, also decreased significantly over the five days of water deprivation (day 3, 70 ± 12% vs. day 8, 51 ± 7%; t_8_ = 3.88, P = 0.0047). Water turnover and the magnitude of selective brain cooling correlated negatively (r^2^ = 0.54, P = 0.024, Fig. 5A), as did water turnover and the proportion of time that animals spent using selective brain cooling (r^2^ = 0.49, P = 0.035, Fig. 5B). A significant, negative correlation was also found between water turnover and the change in body mass of the nine sheep (r^2^ = 0.49, P = 0.037) during the period of water deprivation (Fig. 5C). No relationship was found between the change in body water and change in osmolality (r^2^ = 0.08, P = 0.45; Fig. 5D).

## Discussion

Using stable isotope analysis to measure water turnover in sheep, we have shown, for the first time, that selective brain cooling allows an animal to conserve body water during water deprivation with heat exposure. Total body water comprised ~70% of the body mass of our sheep on the day of water removal (day 3), similar to that previously reported for large herbivores [19], and decreased to about 51% of the body mass following a 5-day period of water deprivation. Our sheep lost 16% of their initial body mass during five days of water deprivation; a decrease similar to that found in Dorper ewes previously exposed to a similar experimental protocol [8], and to Dorper rams that were water-deprived for 4 days [20]. The reduction in the total body water likely was the main cause of body mass loss in our sheep, as has been found for Marwari sheep [21], Sinai goats [22] and Dorper rams [20], with 94% of the total mass loss in the Dorper rams being attributed to water loss. Plasma osmolality of our sheep increased throughout the 5-day period of water deprivation (Fig. 2C), indicating increased water stress over time [23]. The sudden increase in osmolality from the 2^nd^ to the 3^rd^ day of water deprivation (day 4 to 5, Fig. 2C) is consistent with the idea that the rumen can buffer the effects of water deprivation over the short-term, but that the water reservoir of the rumen becomes depleted within a few days of water deprivation [24].

When our sheep had access to water (Fig. 1A, day 1 & 2) brain temperature and carotid blood temperature showed similar profiles, with brain temperature periodically being slightly above carotid blood temperature. Following the removal of water (day 3) both carotid blood and brain temperatures increased. Although arterial blood temperature is the main determinant of brain temperature [25], brain temperature increased by only half as much, on average, as did carotid blood temperature, resulting in an increased magnitude of selective brain cooling with progressive water deprivation (Fig. 1B & Fig. 2A).

Artiodactyls exposed to heat and deprived of drinking water appear to prioritize the conservation of body water by decreasing water loss through evaporative cooling, resulting in their body temperatures increasing, a phenomenon referred to as hypohydration or dehydration-induced hyperthermia [15, 26–28]. If selective brain cooling promotes the conservation of body water, one would expect the increased use of selective brain cooling with water deprivation to result in increased carotid blood temperature. Moreover, one would expect those animals showing the highest magnitude of selective brain cooling to also have the highest carotid blood (core) temperatures when dehydrated. In our sheep, the mean magnitude of selective brain cooling increased during the experimental period, from about 0.2°C when water was available *ad libitum*, to about 0.6°C on the 5^th^ day of water deprivation (Fig. 2A). Concomitantly, carotid blood temperature increased by about 0.6°C during the period of water deprivation, double that previously reported for sheep exposed to a similar experimental protocol [8], but similar to the hypohydration hyperthermia previously found in dehydrated Bedouin goats [15]. We also found a strong relationship between the magnitude of selective brain cooling and carotid blood temperatures. Indeed, selective brain cooling in our sheep explained 61% of the variation in the carotid blood temperatures observed during the period of dehydration. Higher magnitudes of selective brain cooling were therefore associated with higher carotid blood temperatures.

A high carotid blood temperature is not the only factor that drives selective brain cooling [29]. Individual sheep in our study exposed to the same research and husbandry protocol showed considerable variability in the use of selective brain cooling. The variability of selective brain cooling (magnitude and frequency of use) between individuals also increased with continued water deprivation (Fig. 2A & B, day 6 to day 8). These results are likely explained by variation in sympathetic nervous system activation (for review see [1]) between animals with continued water deprivation.

Despite variability between individuals, selective brain cooling, in general, was enhanced with water deprivation. When our study animals had unlimited access to water they used selective brain cooling for 38% of the time. When dehydrated, they used selective brain cooling 61% of the time (Fig. 3B). The proportion of time that our sheep spent using selective brain cooling was not correlated to plasma osmolality, but rather to body water turnover (Fig. 5B), which explained 49% of the variability in the proportion of time spent using selective brain cooling.

The magnitude of selective brain cooling also increased in dehydrated compared to euhydrated states. We found a strong positive correlation between the maximum magnitude of selective brain cooling and both plasma osmolality (Fig. 4A) and the absolute change in osmolality (Fig. 4B), with the osmolality explaining up to 70% of the variability in the maximum magnitude of selective brain cooling. One interpretation of the relationships between selective brain cooling magnitude and osmolality could be that those animals showing the highest levels of selective brain cooling became more dehydrated. However, because the magnitude of selective brain cooling peaked on the fifth day of water deprivation (Fig. 2A) and seven of our nine study animals exhibited maximum magnitudes of selective brain cooling on the last day of water deprivation, the same day that plasma osmolality peaked, we propose that those animals that became most dehydrated showed enhanced magnitudes of selective brain cooling, and as a result had reduced evaporative heat loss drive. Indeed, we found a significant positive correlation between body water turnover and the magnitude of selective brain cooling, which explained 54% of the observed variability, confirming that larger magnitudes of selective brain cooling resulted in smaller changes in body water turnover (Fig. 5A).

Our data support the idea that osmolality enhanced the magnitude of selective brain cooling, but not the proportion of time spent using selective brain cooling. Although hormonal feedback mechanisms following the initiation of selective brain cooling have not been investigated to date, osmolality is likely located upstream of the selective brain cooling-water deprivation sequence. If another variable, such as blood volume contraction, was responsible for enhanced selective brain cooling and the resultant water saving, we would expect osmolality to decrease in those animals showing the largest magnitudes of selective brain cooling. In rats, changes in osmolality as small as 2% are much more effective in altering vasopressin secretion than are changes in blood volume [30]. Although hypovolemia does help to maintain vasopressin release over the longer term, it is not considered to be an effective stimulus for vasopressin release, unless blood volume is reduced by more than 8–10% of the normal blood volume in rats [30] and by more than 10% in sheep [31]. The lack of correlation between changes in body water and changes in osmolality (Fig. 5D) and the plateau in osmolality during the last days of water deprivation (Fig. 2C, day 6 onwards) in our sheep may be explained by the increased electrolyte excretion during water deprivation, as previously documented in ruminants [19, 32] and the decreased food intake with continued water deprivation. Even though we did not quantify daily food intake in our sheep, decreased food intake during dehydration is well documented [33].

We did not measure water turnover in our study animals before the removal of drinking water. Therefore the actual changes in water turnover from a period of euhydration to dehydration could not be quantified in our study. Although artiodactyls do use selective brain cooling when euhydrated, the use of selective brain cooling is enhanced in the absence of drinking water in goats [15], sheep [8], and in free-ranging Arabian oryx [9]. While enhanced selective brain cooling has been reported in dehydrated animals previously [8, 15], to date no one has shown that selective brain cooling results in a saving of body water in dehydrated animals. Our study is the first to show that individuals spontaneously making use of selective brain cooling have lower water turnover rates than conspecifics, living under exactly the same conditions, which use less selective brain cooling.

As shown previously in a study with similar design to ours [8] the threshold temperature for selective brain cooling (~39.4°C) did not change with hydration status. Sheep in that study [8], however, had a much smaller increase in the magnitude of selective brain cooling when dehydrated and heat exposed than sheep in our current study. The lower level of selective brain cooling in those sheep, which possibly resulted from higher sympathetic tone, implies that the animals continued to make use of evaporative water loss to maintain body temperatures [7]. Indeed, carotid blood temperature did not increase during the progressive dehydration of those sheep [8], a result which contrasts with the significant increase in mean carotid blood temperature in our sheep (~ 0.6°C, Table 1) and Bedouin goats [15]. Thus, an increase in core temperature is not a requirement for enhanced selective brain cooling during dehydration. Rather, body water status and hyperosmolality appear to be the primary drivers of selective brain cooling [8, 9]. Hyperosmolality in cats, a species which also can employ selective brain cooling, suppressed evaporative heat loss intra-cranially under conditions of dehydration [34]. Indeed, it is the osmotic pressure of the arterial blood perfusing the brain that stimulates the inhibition of thermoregulatory responses [35]. Inhibition of evaporative heat loss mechanisms, in turn, may contribute to an increase in core temperature.

Differences in temperament and the resultant variation in sympathetic nervous system tone when exposed to the same stressors likely also accounted for the observed increase in variability in selective brain cooling use by individuals with continued water deprivation. The relationship between selective brain cooling and increased sympathetic input (i.e. psychological stress) was first suggested in a study on reindeer [36] and it is now widely accepted that increased sympathetic activity attenuates the use of selective brain cooling in domestic [4, 8, 29] and wild [1, 7, 37] artiodactyls. Merino sheep, for example, abolished selective brain cooling when an investigator entered the holding area [29], while different levels of selective brain cooling in male and female gemsbok have been attributed to the vigilance associated with territorial defense among males [7]. While sympathetic input may alter the actual time that an animal spends using selective brain cooling, the maximum magnitude of selective brain cooling may be mediated by hyperosmolality. Changes in the magnitude of selective brain cooling can, in part, be attributed to variations in the blood flow to the respiratory evaporative heat loss surfaces as well as the evaporative heat loss capacity. For example, when an animal is hyperthermic, there is increased blood flow to the nasal mucosa and other parts of the head compared to during normothermia, while the blood flow to the brain does not change [38, 39]. Increased blood flow to the nasal mucosa, as a result of an increased proportion of the cardiac output passing through arteriovenous anastomoses there [38], could explain the larger magnitude of selective brain cooling when comparing the two hydration periods (Fig. 3A). Whether there is an upper limit to the nasal mucosal blood flow that could take place, or whether no clear upper limit exists, as was found for dogs [40], is not yet known for sheep. The maximum selective brain cooling magnitude recorded in our sheep was comparable to that (1.1°C vs. 1.2°C) previously recorded in the arid-adapted Arabian oryx during summer [9]. Artiodactyls, therefore, may well have similar underlying abilities in terms of the maximum magnitude of selective brain cooling. Even small magnitudes of selective brain cooling are likely to result in the conservation of body water though.

A 50 kg sheep using selective brain cooling for 50% of the time could save an estimated 2.6 liters of water per day (Fig. 5B). Given a daily water intake of 4.2 liters for Dorper ewes in the absence of heat stress [41], such selective brain cooling would result in a ~ 60% saving of normal daily water intake. In European goats artificial cooling of the brain resulted in a water saving of 0.7L per day (or 35% of normal intake) as a result of reduced respiratory evaporative water loss [2]. It was proposed that the water savings in the European goats would be about 70% when water lost through sweating was also taken into account [2], a proportion similar to that saved by our sheep using selective brain cooling spontaneously.

In conclusion, we have measured selective brain cooling and water turnover in nine Dorper ewes deprived of drinking water for a period of five days. We have shown that selective brain cooling increases with water deprivation and that such increases are likely mediated by changes in body fluid osmolality. Increased use of selective brain cooling, either through more frequent use or an increase in the magnitude of selective brain cooling, resulted in increased core temperatures. Although a relationship between selective brain cooling and water conservation has been suggested for the past 20 years [12], ours is the first study to quantify body water savings associated with spontaneous selective brain cooling. We have shown that Dorper sheep can save a substantial amount of their daily water requirements through the use of selective brain cooling. It remains to be investigated to what extent free-ranging artiodactyls, with varying water dependencies and changing seasonal food preferences, use selective brain cooling as a water conservation mechanism in the wild.
